# Effects of Parity and Symmetry on the Aharonov–Bohm Phase of a Quantum Ring

**DOI:** 10.1021/acs.nanolett.1c03882

**Published:** 2021-12-15

**Authors:** Rousan Debbarma, Heidi Potts, Calle Janlén Stenberg, Athanasios Tsintzis, Sebastian Lehmann, Kimberly Dick, Martin Leijnse, Claes Thelander

**Affiliations:** ^†^Division of Solid State Physics and NanoLund and ^‡^Center for Analysis and Synthesis, Lund University, S-221 00 Lund, Sweden

**Keywords:** quantum ring, Aharonov−Bohm effect, quantum dot, symmetry, parity

## Abstract

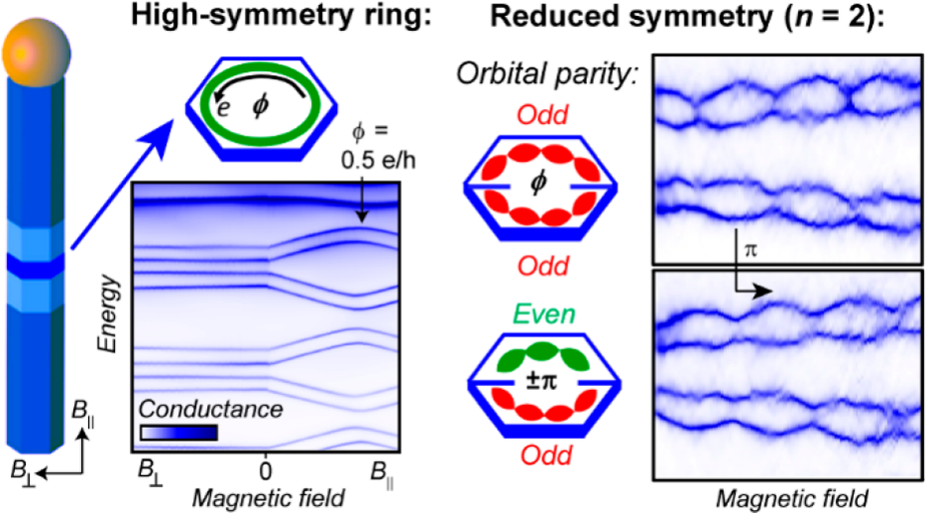

We experimentally investigate the properties of one-dimensional quantum rings that form near the surface of nanowire quantum dots. In agreement with theoretical predictions, we observe the appearance of forbidden gaps in the evolution of states in a magnetic field as the symmetry of a quantum ring is reduced. For a twofold symmetry, our experiments confirm that orbital states are grouped pairwise. Here, a π-phase shift can be introduced in the Aharonov–Bohm relation by controlling the relative orbital parity using an electric field. Studying rings with higher symmetry, we note exceptionally large orbital contributions to the effective g-factor (up to 300), which are many times higher than those previously reported. These findings show that the properties of a phase-coherent system can be significantly altered by the nanostructure symmetry and its interplay with wave function parity.

The particle in a ring represents a standard textbook problem in quantum mechanics^1−3^ and is a case where the topology modifies the properties of the system. Under a varying magnetic field (**B**), a phase-coherent quantum ring displays the Aharonov–Bohm (AB) effect,^4^ where states with different angular momentum signs periodically cross in energy with the enclosed flux. Coherent rings, in both normal and superconducting states, have been important tools when studying how the quantum mechanical phase is affected when a system undergoes changes.^5−13^ By inserting quantum dots (QDs) in one or both arms of a ring, the phase-coherence of electron tunnelling can be studied^5,9,11^ as well as the effect of single spins on the phase difference between superconducting macroscopic states.^12,13^

In this work, we use quantum rings to study the interplay of the wave function parity with the symmetry of a system. While an ideal quantum ring has perfect and infinite rotational symmetry, any real ring-like structure will display imperfections. In a number of theoretical works on semiconductor-based quantum rings, it was shown that a reduction in the symmetry of a ring (or tube) results in states undergoing avoided crossings as a function of the magnetic field, leading to energy gaps in the spectrum.^14−21^ In essence, a system with an *n*-fold rotational symmetry of the confinement potential should show orbital states in groups of *n* separated by gaps close to which the orbital angular momentum approaches zero.^15^ Away from these energy gaps, the states are ring-like and show a significant orbital angular momentum.

Pham et al.^22^ recently studied hexagonal rings formed by the presence of indium adatoms on an InAs surface and found a lifting of orbital degeneracies consistent with a reduced symmetry. Zhu et al.^14^ calculated the properties of quantum rings with two symmetric barriers (*n* = 2) and predicted an electronic structure of orbital states grouped in pairs. This particular case is, however, very different from higher ring symmetries^15^ in that it has no ring-like states at zero magnetic flux (ϕ) for combinations of orbitals with the same parity, whereas different parity combinations are predicted to be ring-like. At half a flux quantum, ϕ = 0.5 h/e, the effect of parity was predicted to reverse, resulting in a π-phase shift in the AB effect. Indeed, the ring-like nature of such even–odd parity combinations at ϕ = 0 was recently experimentally confirmed by Potts et al.^23^ and was explained by a cancellation of the overlap integrals at the two barriers.

In this work, we have experimentally and theoretically examined the AB effect in structures similar to those in ref (23), where phase-coherent quantum rings form near the surface of nanowires with epitaxially defined QDs. Using transport measurements, we find AB flux periodicities corresponding to rings that have an approximately 10 nm smaller radius than the nanowire, consistent with a surface accumulation layer of electrons.^24^ We realize one-dimensional rings with different symmetries and observe a huge orbital contribution to the electron *g*-factor for high symmetry cases. For the lowest symmetry (*n* = 2), corresponding to a pair of QDs connected in two points, we confirm that orbitals are grouped pairwise in a **B**-field. By controlling the orbital parity in each QD, we also find the predicted π-phase shift in the AB oscillations, which stems from a **B**-field periodic reversal in the orbital requirements for when the overlap integrals cancel. The experimental results were reproduced with tight-binding calculations where the different ring symmetries were studied under electric and magnetic fields.

Two different types of QD ring structures (A and B) are investigated in this work, both of which are formed by controlling the crystal structure of InAs nanowires during epitaxial growth. Sample A has a QD of pure InAs, whereas B has an additional thin outer shell of InAsSb. Surface-related band bending in InAs^25^ and InAsSb^24^ is key to the formation of the ring-like states that we observe. The interior of the QD has no filled states due to the strong confinement, while electrons can still accumulate near the surface.^23^ However, the resulting ring-like potential around the nanowire circumference is sensitive to perturbations, especially in a low carrier concentration limit, resulting in localized electron pockets.^26^ The purpose of including two types of samples in this study, with a second sample having Sb in the InAs surface layer, is twofold, first to enhance the surface electron concentration as predicted from an Sb-induced conduction band bowing^27^ and thereby screen effects of symmetry-breaking defects and second to further increase the spin–orbit interaction (SOI).

Nanowires were grown using metalorganic vapor-phase epitaxy from Au seeds arranged in periodic patterns, where the crystal structure of InAs was controlled as reported in ref (23). They were designed to have long outer contact segments with zinc blende (ZB) structure with a high carrier concentration. These segments connect to a pair of tunnel barriers with wurtzite (WZ) structure, which in turn sandwich a very short (4–5 nm) middle ZB segment to provide strong confinement there (QD type A, Figure 1b). The growth of the second sample (QD type B, Figure 1a and c) was terminated with an InAsSb growth step, which added an approximately 4 nm shell of InAsSb and an axial segment near the top of the nanowires compared to the reference NWs.^28^ Energy-dispersive X-ray spectroscopy performed near the surface of the zinc blende segments gave an InAs_0.8_Sb_0.2_ composition. For growth details, see the Supporting Information. Source and drain contacts to the nanowires were fabricated using Ti/Al and Ti/Au (type A sample) or Ni/Au only (type B).^26^ The contact layout also included side-gating electrodes to control the electrostatic potential within the central ZB segment. Transport measurements were carried out in a dilution refrigerator at an electron temperature <100 mK, where the magnetic field direction and strength were controlled with a vector magnet.

**Figure 1:**
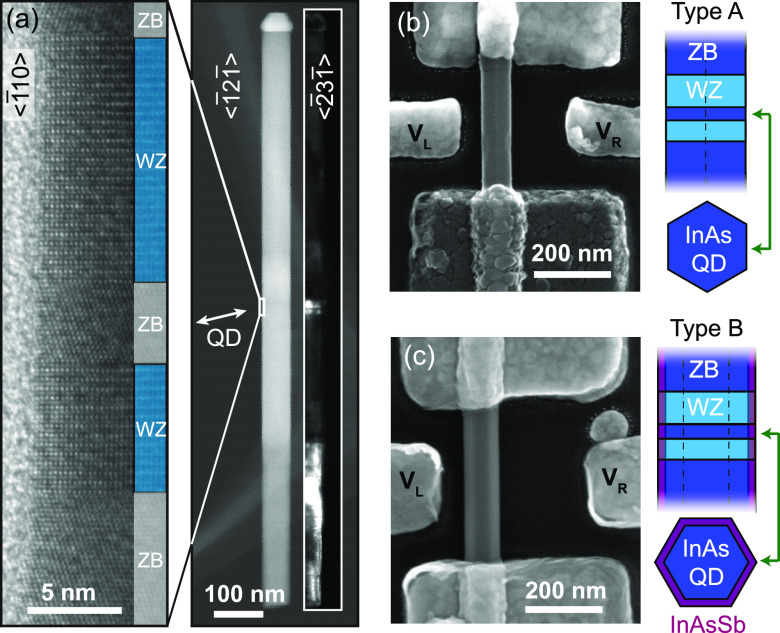
(a) High-resolution, high angle annular dark field, and dark field (inset) transmission electron microscopy (TEM) images of a type B nanowire showing the wurtzite (WZ) barriers around the zinc blende (ZB) quantum dot segment. (b) Scanning electron micrograph (SEM) of the type A InAs nanowire device, with source-drain and side-gating electrodes. (c) SEM of the type B InAs–InAsSb core–shell nanowire device.

We start by presenting the results of the type B sample, which could be electrostatically tuned to become a high symmetry ring. The overview plots in Figure 2a show the conductance in the QD as a function of the two side-gate voltages, *V*_L_ and *V*_R_. The lines here correspond to transport through the QD ground state, where the electron number increases in steps of one for each line at higher *V*_L,R_ going from approximately 18 to 36. We note that each line pair represents a spin-degenerate orbital separated by the QD charging energy, *E*_*C*_, and that two line pairs here frequently form groups of four states. The absence of a honeycomb structure and the almost similar slopes of the conductance lines support that transport occurs trough a single QD in this regime.^7^ Also shown in Figure 2a is the effect of a **B**-field of 0.2 T applied parallel (*B*_||_) to the nanowire, which significantly changes the state energies.

**Figure 2:**
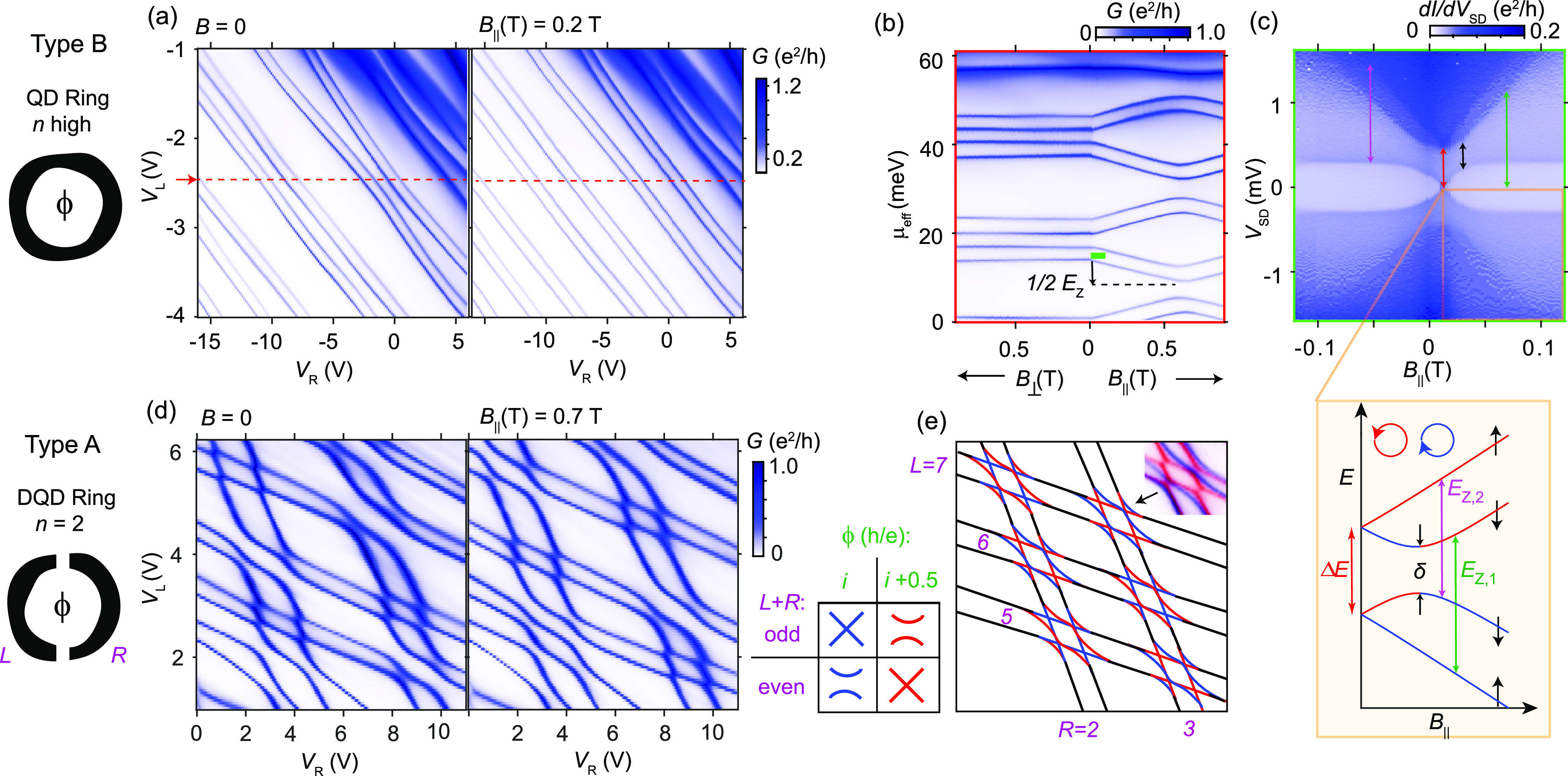
Effects of **B**-fields on orbital and spin states in QD rings with different symmetries (*n*). (a) Conductance of a QD ring (sample type B) as a function of *V*_L,R_ at **B** = 0 (left) and *B*_||_= 0.2 T (right) for a back-gate voltage, *V*_BG_ = 3 V, and source-drain voltage, *V*_SD_ = 0.1 mV. (b) Conductance along the red line in panel a vs *B*_⊥_ and *B*_||_. (c) Differential conductance vs *B*_||_ in the one-electron regime along the green bar in panel b. The inset shows schematically how the four nearly degenerate spin and orbital states evolve with *B*_||_, where it is possible to identify *E*_Z_ and extract *g*_orb_ from *E*_Z_ = *g*_orb_μ_B_*B*_||_. (d) Conductance of a double QD ring (sample type A) for **B** = 0 and *B*_||_ = 0.7 T at *V*_BG_ = 0 V and *V*_SD_ = 0.5 mV. (e) Schematic for six consecutive orbital crossings at ϕ = 0 and 0.5 h/e. Orbital parity and threaded flux (*i*, integer) determine whether each crossing is exact or avoided. The small inset shows an overlay of data from panel d at the two fields.

Figure 2b shows the **B**-field evolution of the conductance associated with ground-state transport for fields applied both parallel (*B*_||_) and perpendicular (*B*_⊥_) to the nanowire axis, which was obtained along the indicated gate vector in Figure 2a. The energy axis (μ_eff_) is derived from an extraction of the gate-lever arm of Coulomb diamonds along that vector (Supporting Information). We observe that the level structure and **B**-field evolution in Figure 2b is similar to that found in carbon nanotube QDs, where spin and orbital degrees of freedom result in a fourfold degeneracy of states and where the energy splitting *E*_Z_ = g*μ_*B*_**B** strongly depends on the **B**-field direction. This anisotropy comes from the effective *g*-factor given by g* = *g*_spin_ ± *g*_orb_. Here, *g*_spin_ is related to the spin magnetic moment, whereas *g*_orb_ is related to the orbital magnetic moment, which couples only to the **B**-field component that threads the ring. From the shift in ground state energies we obtain *g*_orb_ = 290 as well as an upper limit for |*g*_spin_| of approximately 15.

These very large values result from the scaling of *g*_orb_ with the orbital quantum number *l* according to *2(m*_0_/m*)*l*, where m* is the effective electron mass.^29^ We note that the QD here holds roughly 25 electrons, which would imply *l ≈* 6 in a perfect 1D ring picture. The strong confinement from the axial crystal phase segments is a key reason to why it is possible to track orbital states over such large energies (>10 meV). In carbon nanotube QDs, the corresponding axial confinement is generally much weaker, resulting in low-energy axial excitation modes.

At *B*_||_ = 0.6 T, the ground states change as orbitals with a different angular momentum sign become more energetically favorable. From the orbital crossing we can extrapolate an AB periodicity of *ΔB* = 1.2 T, which corresponds to a ring diameter of *D*_AB_ = 66 nm using *ΔBA* = h/e, where *A* is the enclosed loop area. Based on a physical NW diameter of 90 nm (Figure 1c), we note that the ring radius is about 10 nm smaller than the NW radius.

Figure 2c shows the excited state spectrum for small values of *B*_||_ along the green bar in Figure 2b. We refer to this as a single-electron regime, thus neglecting filled orbitals. The expected fourfold degeneracy of spin and orbital states at **B** = 0 is broken here by the combination of an SOI and disorder (δ); thus, the electronic structure is very similar to that of carbon nanotube QDs.^30^ The consequence of the large *g*_*or*b_ value is that the two lowest energy states already have the same orbital angular momentum sign for *B*_||_ > 30 mT. We can also extract the effective *g*-factors from the excited-state spectrum using the splitting of the Kramer’s pairs in Figure 2c (*E*_Z,1_ and *E*_Z,2_ in the inset). Assuming a negative *g*_spin_, the effective *g*-factors corresponding to *E*_Z,1_ and *E*_Z,2_ are 275 and −300, respectively, which are consistent with *g*_spin_ = −12 and *g*_orb_ = 290 extracted from the shift in the ground state energies in Figure 2b.

The avoided crossing, δ = 330 μeV, indicated in Figure 2c is a consequence of a disorder that couples electrons of the same spin from different orbitals. Based on an energy gap *ΔE* = 470 μeV at **B** = 0 and *ΔE*^2^ = δ^2^ + SOI^2^, we extract SOI = 330 μeV. We note that this value is larger than the corresponding value extracted in a similar structure consisting of InAs only.^23^ This may be related to a non-negligible tail in the electron distribution that resides in the thin InAsSb shell^31^ where SOI is strong.

Next, we investigate a nanowire of type A, which has two doubly connected QDs that result in a reduced ring symmetry (*n* = 2) with important consequences for the magnetic-field-dependence of the orbital states. Plotting the conductance in this sample as a function of *V*_L_ and *V*_R_ reveals sets of lines with two different slopes (Figure 2d). The conductance pattern is explained by transport through a double QD, which is parallel-coupled to a source and a drain.^26,32^ The two side-gates have different electrostatic couplings to each QD, and orbitals belonging to different QDs therefore cross in energy and interact. Similar to the case in Figure 2a, each orbital is spin-degenerate and therefore has a conductance replica at higher energy, separated by a charging energy.

The numbering of the orbital crossings indicated in the cartoon in Figure 2e is based on an overview measurement provided in the Supporting Information. We will focus on six consecutive orbital crossings for which the orbital structure and behavior with the electric field are predictable and where the electron numbers range from 12 to 20. However, we note that the double QD is not ideal over a wider span of gate voltages such that the selective gate coupling to some orbitals is not constant over the entire measurement range.

When orbital pairs align in energy, it is clear that half the crossings are exact, whereas the other half are seemingly strongly tunnel-coupled, as indicated by the avoided level crossings. This observation is in line with the findings in ref (23), where exact crossings are explained by a cancellation of the hybridization energy due to the different signs of the overlap integrals at the two tunnel barriers within the ring. This only occurs for cases where an odd orbital aligns in energy with an even orbital, corresponding to a difference in parity. In contrast, for even–even and odd–odd combinations with the same parity, the overlap integrals do not cancel, and an energy gap forms in a double QD ring. Due to this parity requirement, the conductance lines in Figure 2d form an alternating checkerboard pattern of the two crossing types. An identical pattern was predicted by Zhu et al.,^14^ where orbital energies were plotted as a function of the angle between the two barriers in the ring, thus varying the relative sizes of the ring segments to fit different excitations. In our case, we believe that the ring segment sizes are approximately fixed and that the wave function parities are controlled by electrostatic shifts in energy.

Interestingly, the pattern is completely reversed upon the application of a magnetic field component *B*_*||*_ = 0.7 T through the ring that corresponds to half a magnetic flux quantum, ϕ = 0.5 h/e (as shown later). Orbital crossings that were exact are now avoided, and vice versa, such that the parity requirement is the opposite. However, we note that away from orbital crossings the individual QD orbitals are not significantly shifted by the **B**-field, as was the case for the QD of type B, indicating that these localized states have no, or small, orbital momentum contribution to g*. This is highlighted in the cartoon in Figure 2e and exemplified by the inset representing a local overlay of two measurements from Figure 2d.

To explain how a reduced ring symmetry gives rise to the experimental results, we calculated the evolution of states as a function of the magnetic field for the cases of perfect, four-barrier, and double-barrier ring symmetries, as shown in Figure 3a–c, respectively. The theoretical calculations involved tight-binding discretization of a circular 2D QD, which was used to simulate the different ring types with the inclusion of appropriate periodic potentials as well as an external electric field. For details on the model, see the Supporting Information.

**Figure 3:**
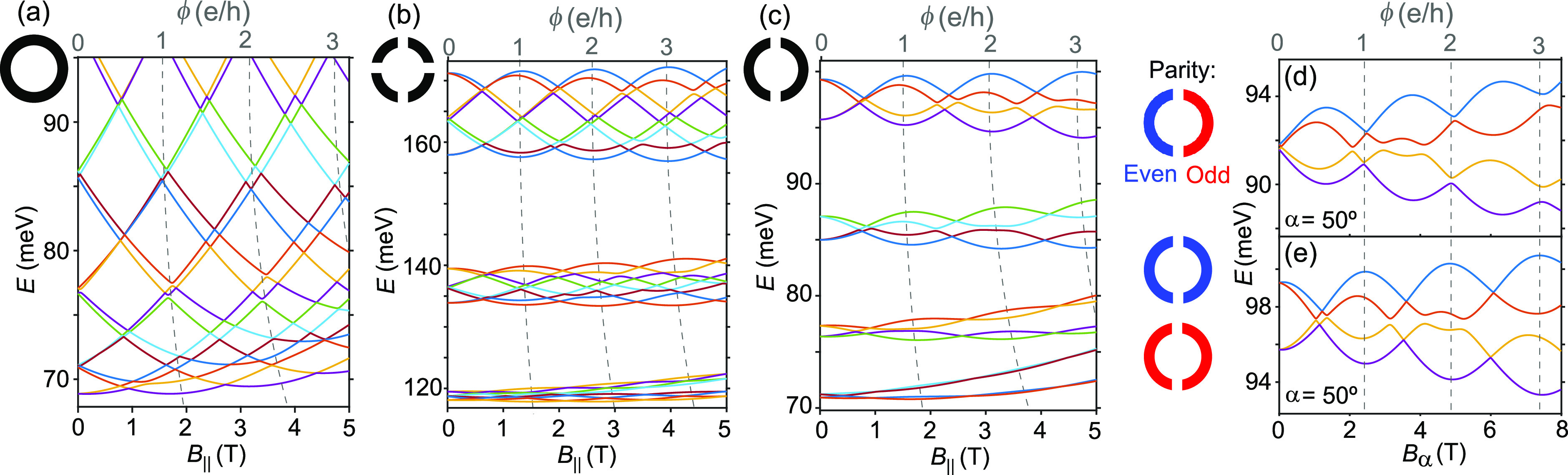
Calculation of electron energies as a function of **B**-field for quantum rings with different symmetries. (a) A perfect quantum ring in a 1D limit, here modeled as a 2D ring in an energy range with no transverse excitations. (b) Ring with four barriers (*n* = 4), which give rise to large avoided crossings and a fourfold grouping of orbitals. (c) Ring with two barriers (*n* = 2), which has no ring-like states (⟨*L*⟩ ≈ 0) at **B** = 0. (d and e) Effect of the relative orbital parity for *n* = 2, where a difference in parity results in a π-shift in the AB effect compared to the same parity. We note that the value of ϕ is inferred from the orbital crossings with a magnetic field, which in a non-1D system depends on the energy.

We first note that a symmetry reduction introduces a grouping of orbital and spin states with the magnetic field according to the symmetry, as previously predicted.^15^ Notably, no ring-like states are observed at **B** = 0 in the lowest (*n* = 2) symmetry case when both ring-halves have the same parity (Figure 3c). However, under an electric field, which provides a further symmetry-breaking effect, a different orbital parity can be introduced. Figure 3d and e show the evolution of orbital pairs where the half-rings have different (d) and the same (e) orbital parities (orbitals 4,3 in panel d and orbitals 4|,4 in panel e). It is clear that the AB pattern experiences a π-phase shift such that ring-like states with large ⟨*L*⟩ appear at ϕ = 0 when the parity is different and also at ϕ = 0.5 h/e for the case of the same parity. For the coming comparison with the experiment, we here used a nonparallel direction of the **B**-field (α = 50° from *B*_||_), which resulted in a weaker orbital splitting.

In the experiment, the conductance lines are separated not only by the energies of the single-particle orbitals but also by a charging energy *E*_C_. Therefore, we next introduce such an additional splitting *E*_C_ between the calculated single-particle orbital energies (this corresponds to the result of the constant interaction model for a QD). First, the ideal ring case is shown in Figure 4a, whereas in Figure 4b we replot the experimental data for the type B nanowire from Figure 2b as a comparison. We note that overall the experimental ring looks very similar to the ideal ring, but with slightly rounded features at *B*_||_ = 0.6 T indicative of small avoided crossings. Since we cannot induce uniform changes in the ring potential with only two side-gates, it is unclear whether the experimental ring has an inherent sixfold symmetry from the hexagonal cross-section (Figure 1c). However, we note that some symmetry-reducing barriers must be present as we observe similar ⟨*L*⟩ values in Figure 4a and b in a situation where the experiment has more electrons than the model.

**Figure 4:**
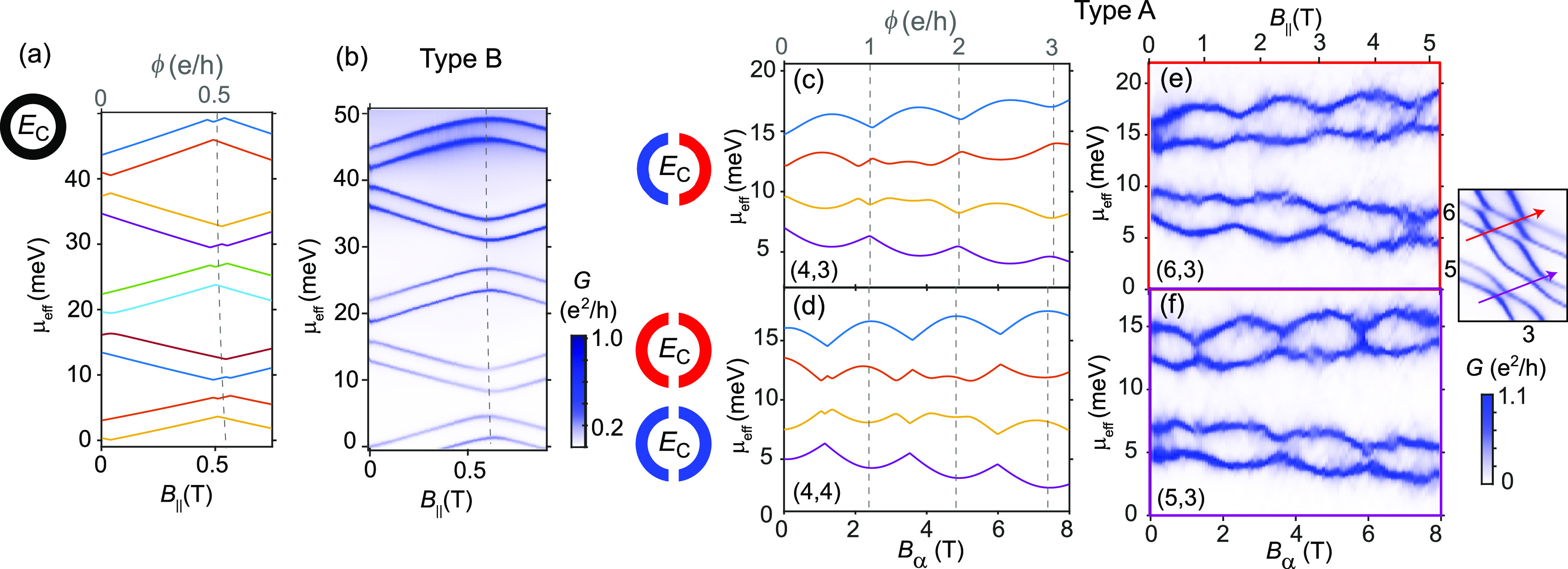
(a) Evolution of states with parallel **B**-field in a perfect ring (*D*_AB_ = 66 nm), including a charging energy, *E*_C_, extracted from the corresponding experiment. (b) Replotting of data in Figure 2b as a comparison, where we find that the QD can behave like a high-symmetry ring within a range of electric fields. (c and d) Charging energy (*E*_C_) added to the energies in Figures 3d and e, respectively, for a double-barrier ring. (e and f) Experiment showing the evolution of ground state energies with *B*_α_ when passing through two consecutive honeycombs of different relative orbital parity as shown in the inset (cropped from Figure 2d). We note a π-phase shift resulting from the change in relative parity, and the presence of ring-like states at **B** = 0 for the case of different orbital parity. A **B**-field vector misaligned from the NW long axis was used (α = 50°), which allows access to stronger fields in the setup. From the shift in GS energy, we can extract a maximum |g*|= 80.

Next, we go back to the type A nanowire and compare experiments with simulations that include *E*_C_, as shown in Figure 4c–f. Similar to Figure 4b, we plot the conductance of the ground states as a function of μ_eff_ and the **B**-field; here, however, we are comparing measurements through two consecutive honeycombs. The experiments confirm the predicted π-phase shift in the AB effect when changing the relative orbital parities. In both cases we also note that ring formation is periodic in *B*_α_ (α = 50° from *B*_||_) with a period of *B*_α_ = 2.3 T (*B*_||_ = 1.5 T), which corresponds to a ring diameter of *D*_AB_ = 59 nm. With a physical diameter of 80 nm, we note a similar 10 nm difference in radius as that for the type B case, which points to a substantial weight of the wave function rather close to the NW surface. This direct correlation of ring and nanowire diameters, and a *D*_AB_ that seems to be independent of electron number within the range of Figure 2b, supports the presence of a surface electron accumulation layer.^24^

In conclusion, we have engineered one-dimensional rings in QDs where electrons gain an exceptionally large orbital angular momentum. Due to a strong phase coherence in the system, uncommon for nonsuperconducting rings, we can probe theoretical predictions on the effects of symmetry and parity of a quantum ring. In agreement with the predictions, we find that rings composed of two doubly connected QDs experience an Aharonov–Bohm period similar to that of a sample with a higher symmetry but with an evolution of states with **B**-field that is very different. The resulting orbitals are grouped according to the symmetry (*n* = 2), with a phase determined by the orbital parities of the QDs. By modifying the material of the ring, here attempted by introducing an outer shell with a reduced band gap, an even more efficient manipulation of electronic states should be possible.

## Supporting Information Available

Description of the theoretical model used in the tight binding calculation; growth details and the morphological, structural, and compositional analysis of the nanowires; additional electrical measurements; and description of the calculation of μ_eff_ (PDF)
