# Supplementary material for: Effects of Parity and Symmetry on the Aharonov–Bohm Phase of a Quantum Ring
Source: Nano Lett. 2021 Dec 15;22(1):334–9. doi: 10.1021/acs.nanolett.1c03882 (PMC8759086; doi:10.1021/acs.nanolett.1c03882)
Supplement: Supplementary file 1 — nl1c03882_si_001.pdf [file nl1c03882_si_001.pdf]

Supporting Information for:

Effects of parity and symmetry on the Aharonov-Bohm phase of a quantum ring

Rousan Debbarma,[1] Heidi Potts,[1] Calle Janlén Stenberg,[1] Athanasios Tsintzis,[1] Sebastian Lehmann,[1] Kimberly Dick,[1,2] Martin Leijnse,[1] Claes Thelander[1]

[1]Division of Solid State Physics and NanoLund and [2]Center for Analysis and Synthesis, Lund University, S-221 00 Lund, Sweden

1. Theoretical Model

Considering the z direction to be along the NW axis, the sections where the QD and the DQD are located are modeled with 2D disks in the xy plane. In order to model the Type B ring QD a dome-shaped potential is added with the maximum located at the center of the disk, rendering the disk boundary more energetically favorable for electrons. To simulate the Type A DQD system a potential barrier with a Gaussian profile in the x coordinate is additionally included along the disk diameter in the y direction, separating the ring in two half-rings, left (L) and right (R). The DQD system is depicted in Fig. S1.

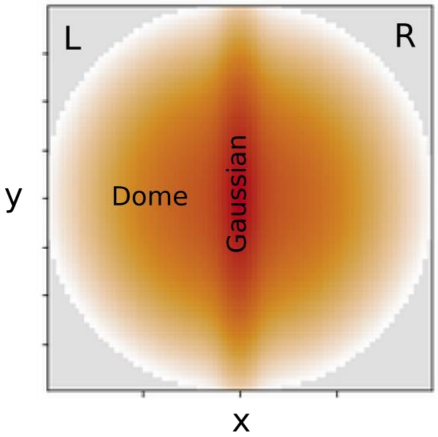

Figure S1: Model of the Type B DQD system. The NW axis is in the z direction and the QD area is considered to be a disk in the xy plane. A dome-shaped potential peaking at the center of the disk creates a ring-like potential landscape for electrons and a Gaussian x-dependent potential barrier along the disk diameter in the y coordinate separates the left (L) from the right (R) QD.

In the effective mass approximation and for a finite magnetic field the one-electron Hamiltonian of the system is

$$H = H_0 + H_Z + H_{SO} + V(\mathbf{r}), \tag{1}$$

where H_0 is the free particle Hamiltonian, H_Z is the Zeeman term, H_{SO} the Rashba spin-orbit (SO) term and $V(\mathbf{r})$ the electrostatic potential. Introducing each term separately,

$$H_0 = \frac{(\mathbf{p} + e\mathbf{A})^2}{2m^*}, \tag{2}$$

$$H_Z = \frac{g^*_{\text{spin}}}{2} \mu_B \boldsymbol{\sigma} \cdot \mathbf{B}, \tag{3}$$

$$H_{\mathrm{SO}} = \frac{\alpha_{\mathrm{SO}}}{\hbar}\big(\mathbf{e} \times (\mathbf{p} + e\mathbf{A})\big) \cdot \boldsymbol{\sigma}\,, \tag{4}$$

where $\mathbf{p} = -i\hbar\nabla$ is the momentum operator, m^* the effective electron mass, e the elementary charge ($e > 0$), $\hbar$ the reduced Planck's constant, g^*_{spin} the effective g-factor, μ_{B} the Bohr magneton, α_{SO} the SO strength, $\boldsymbol{\sigma}$ the Pauli vector and $\mathbf{e} = (0, 0, 1)$ the direction of the built-in electric field giving rise to the SO term. The applied magnetic field is $\mathbf{B} = (0, B_{\mathrm{y}} \sin\alpha,\ B_{\mathrm{z}} \cos\alpha)$**,** the vector potential is taken to be $\mathbf{A} = (zB_{\mathrm{y}} \sin\alpha,\ xB_{\mathrm{z}} \cos\alpha\,, 0)$ and the direction of the magnetic field in the yz plane is controlled by α. Since the model is strictly 2D we set $z = 0$ and finally obtain $\mathbf{A} = (0,\ xB_{\mathrm{z}} \cos\alpha\,, 0)$. The electrostatic potential $V(\mathbf{r})$ includes the dome-shaped potential and the Gaussian barrier splitting the ring in half. The dome-shaped potential is given by

$$V_{\mathrm{dome}}(\mathbf{r}) = \kappa(R^2 - x^2 - y^2), \tag{5}$$

where κ is a parameter controlling the maximum dome height and R is the NW radius. The Gaussian barrier is given by

$$V_{\mathrm{G}}(x) = \lambda \exp(-bx^2), \tag{6}$$

where λ, b are parameters controlling the Gaussian shape and size. The values of the parameters appearing in the above equations can be found in Table S1.

**Parameter**	**Value**
m^*	0.026 m_{e}
g^*_{spin}	10
α_{SO}	16 meV $\cdot$ nm
R	40/46 nm
κ	0.0655 meV $\cdot$ nm^{-2}
λ	78.6 meV
b	0.05 nm^{-2}

Table S1: Parameters used in the TB calculations. m_{e} is the electron mass and the values of m^* corresponds to zinc blende (ZB) InAs. g^*_{spin} is taken somewhat smaller than the InAs bulk value ($|g^*_{\mathrm{spin}}| \sim 14.9$) to account for confinement effects. The given NW radii (R) correspond to Type A/B samples. An exception is made for Figure 3(a) for which $R = 40$ nm. The values of the parameters $\alpha,\ \kappa,\ \lambda,\ b$ were tuned to match experimental results. For the Type B sample $\lambda = 0$.

The above Hamiltonian was discretized in a square lattice of circular shape (see Fig. S1) with lattice constant $d = 1$ nm. The python package Kwant[1] was used to build and diagonalize the tight-binding (TB) matrix.

The evolution of the electron energies with an increasing magnetic field depicted in Figures 3(a, b, c) in the main paper can be readily calculated. For the perfect ring case in Figure 3(a) no Gaussian-shaped barriers are included, while for Figure 3(c) one Gaussian-shaped barrier is included along the disk diameter in the y direction, as shown in Fig. S1. For Figure 3(b) an additional Gaussian-shaped barrier is included along the disk diameter in the x direction.

For the Type A DQD modeled in Figure 3(c) the rotational symmetry is broken and the states of the system are not ring states at $\mathbf{B} = 0$. In order to retrieve ring states an asymmetry in the DQD system is introduced so that the energy of an even (odd) orbital in dot L is equal to the energy of an odd (even) orbital in dot R. We model this asymmetry by introducing a linear x-dependent detuning potential of the form

$$V_{\Delta} = \Delta \frac{x}{R} , \tag{7}$$

quantified by the *detuning* Δ. In the experiment, the detuning is controlled by the side-gate voltages V_{L} and V_{R}. The coordinate system is such that for $\Delta > 0$ the energies of the L dot ($x < 0$) states decrease and the energies of the R dot ($x > 0$) states increase. The DQD energies with respect to Δ for $\mathbf{B} = 0$ are plotted in Fig. S2(a). For $\Delta \approx 11.8$ meV the fourth orbital in the L dot is aligned with the third orbital in the R dot and the (4,3) crossing is marked with the red rectangle. The states are doubly degenerate and thus four states in total are involved in the crossing. The magnetic field evolution of these four states for $\Delta \approx 11.8$ meV is shown in Figure 3(d) in the main paper, where we note that the magnetic field direction is at an angle $\alpha = 50°$ with respect to the z direction. The ring-like features of these states are illustrated in Fig. S2(b) which is a close-up of Figure 3(d) for magnetic fields ($|\mathbf{B}| = 0 - 0.4$ T). At $|\mathbf{B}| = 0$ the states only split by the SO interaction and each state is two-fold degenerate. Effective g-factors of $g_1^* = 78$ and $g_2^* = 92$ can be read-off from the linear slope at small $|\mathbf{B}|$ for the higher and lower energy pair of states respectively. Since $g_{\mathrm{spin}}^* = 10$ these large g-factors are attributed to orbital contributions which imply ring-like states. The situation is very different if one focuses on the (4,4) (anti-)crossing at $\Delta = 0$, marked with the blue rectangle in Fig. S2(a). The magnetic field evolution for the four states involved is shown in Figure 3(e) in the main paper and a close-up for small fields can be seen in Fig. S2(c). In this case the states are not ring-like for small magnetic fields and for the extracted effective g-factors we have that $g_1^* \approx g_2^* \approx g_{\mathrm{spin}}^*$, which indicates the absence of orbital contributions.

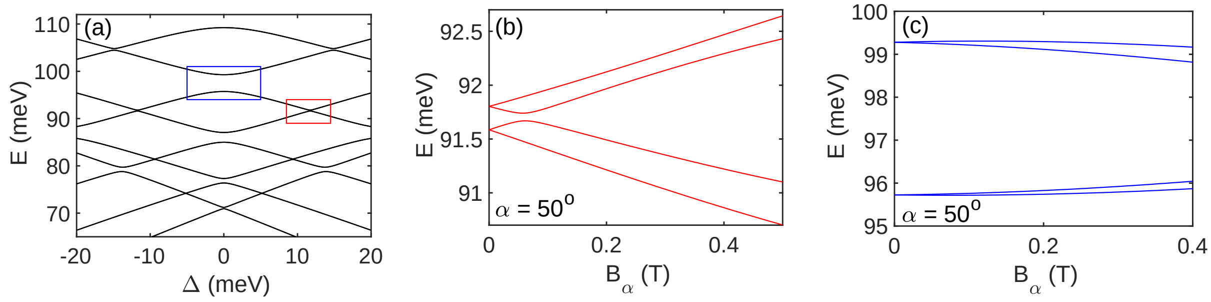

Figure S2: (a) Energy levels of the DQD as a function of detuning Δ at $\mathbf{B} = 0$, where each state is twofold degenerate. The (4,3) crossing is depicted by the red box at $\Delta = 11.8$ meV, while the blue box marks the (4,4) crossing at $\Delta = 0$ (b) The magnetic field dependence of the (4,3) states at a fixed detuning Δ = 11.8 meV. (c) The magnetic field dependence of the (4,4) states at a fixed detuning Δ = 0.

2. **Experimental**

a. Growth details: InAs nanowires and InAsSb

The synthesis of Type A InAs nanowires using metal organic vapor phase epitaxy in an Aixtron 3x2´´ close coupled showerhead reactor is described in detail in Potts et al.[2] For Type B nanowires the InAs core nanowire growth was similar to the synthesis of Type A nanowires with only the growth time of the second Zb segment being cut to 4 minutes. The $InAs_{1-x}Sb_x$ shell growth followed the procedure published by Dahl

et al.[3] and was initiated by lowering the set temperate to 450°C in an AsH_3/H_2 atmosphere before introducing the precursors with corresponding molar fractions of $\chi_{\mathrm{TMIn}} = 2.6\mathrm{E}-06$, $\chi_{\mathrm{AsH_3}} = 1.3\mathrm{E}-04$, and $\chi_{\mathrm{TMSb}} = 3.1\mathrm{E}-04$, respectively. The $InAs_{1-x}Sb_x$ shell growth time was set to 8 minutes.

b. Morphological, structural, and compositional analysis:

For detailed morphological, structural, and compositional analysis electron microscopy (EM) was carried out in a Hitachi SU8010 (SEM) and a JEOL 3000F (TEM) both equipped with field emission guns and operated at 15 and 300 kV, respectively. For compositional analysis, energy-dispersive X-ray spectroscopy (EDS) data was extracted from maps acquired in high angle annular dark field scanning transmission electron microscopy mode (HAADF-STEM). As displayed in Fig. S3, dark field imaging along a $\langle\bar{2}3\bar{1}\rangle$ direction served as a guide for location of the QD position along the axis of the nanowire for HAADF-STEM analysis in a $\langle\bar{1}2\bar{1}\rangle$ direction (Fig. S3 a,b). This was necessary since the distinction between WZ and ZB is not possible in HR-TEM when imaging along this crystallographic direction. The nanowire core to shell interface forms on {110}-type facets meaning that the interface can only be imaged when aligning the nanowire with a $\langle\bar{1}2\bar{1}\rangle$ direction parallel to the incoming electron beam.

Sum X-ray spectra were extracted from the full nanowire cross-section at positions above (green), below (blue), and at the position of the WZ barriers and the ZB QD (red) as indicated by the dotted rectangles in Fig. S3 b. Those spectra were smoothed and normalized to the maximum intensity of the In $\mathrm{L}\alpha_{1/2}$ emission at around 3.29 keV before a reference spectrum of an InAs nanowire, normalized as well to the In $\mathrm{L}\alpha_{1/2}$ emission, was subtracted. The residual, smoothed spectra are given in Fig. S3 c in the energy range covering Sb related $\mathrm{L}\alpha_{1/2}$ (~3.6 keV), $\mathrm{L}\beta_1$ (~3.8 keV), and $\mathrm{L}\beta_2$ (~4.1 keV) emission lines. A similarly treated spectrum of an InSb nanowire is also shown (normalized to the In $\mathrm{L}\alpha_{1/2}$ emission and subtracted by the normalized InAs nanowire reference spectrum), which confirms the presence of Sb for the Type B nanowire.

For an analysis more sensitive to the shell, we have extracted spectra from the estimated shell position on one side of the nanowire only as indicated by the solid line rectangles indicated in Fig. S3 b. Those spectra were smoothed, normalized to the maximum intensity of the In $\mathrm{L}\alpha_{1/2}$ emission line, and are displayed in Fig. S3 d together with the reference spectra of InAs and InSb nanowires, both of which are normalized as well to the In $\mathrm{L}\alpha_{1/2}$ emission. The shoulders at the position of the Sb related $\mathrm{L}\alpha_{1/2}$ emission line (~3.6 keV) are clear indications of Sb incorporation in the shell. The tendency of a lower signal for the shell formed at the position of the WZ barriers, here also including the thin ZB QD, (red solid rectangle) indicates a similar tendency as reported earlier in Dahl et al.[3] We find compositions of $InAs_{0.80(2)}Sb_{0.20(2)}$ on ZB and $InAs_{0.87(2)}Sb_{0.13(2)}$ on WZ, respectively, using the Oxford Inca software and the characteristic lines of the involved elements (In $\mathrm{L}\alpha_1$, Sb $\mathrm{L}\alpha_1$, and As $\mathrm{K}\alpha_1$).

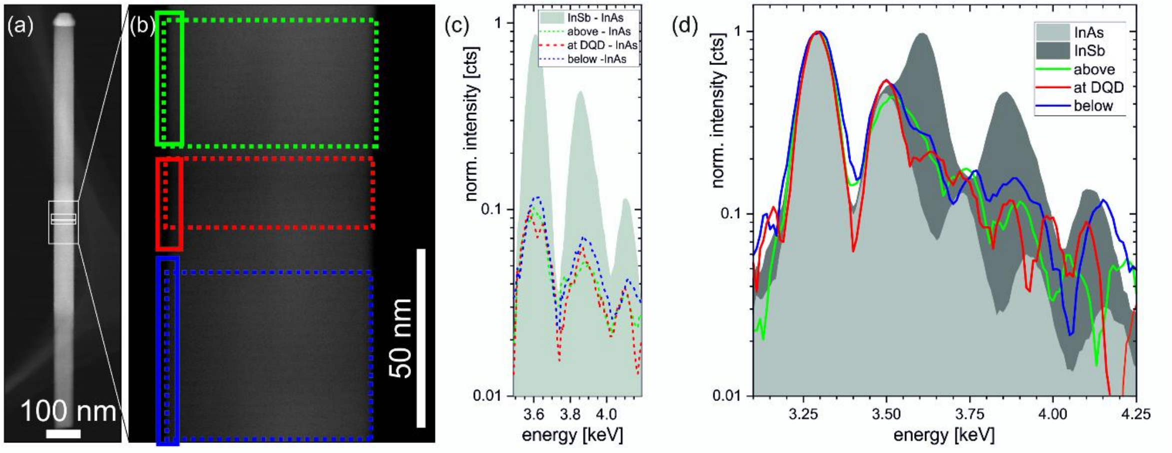

Figure S3: (a) HAADF-STEM image taken along a <112>-type direction with (b) a higher resolution inset of the position of the QD. Color-coded squares indicate where EDS sum spectra were extracted which are displayed in (c) and (d). (c) Detail of the EDS sum spectra, after normalization to the In $\mathrm{L\alpha_{1/2}}$ emission and subtraction of the In $\mathrm{L\alpha_{1/2}}$, $\mathrm{L\beta_{1/2}}$, and $\mathrm{L\gamma_1}$ contribution, showing the extracted Sb $\mathrm{L\alpha_{1/2}}$, $\mathrm{L\beta_1}$, and $\mathrm{L\beta_2}$ emission lines, respectively. The data was extracted from the nanowire at positions above (green - dotted), below (blue - dotted), at the WZ barriers including the QD (red - dotted) and is shown together with a reference spectrum (grey). This reference spectrum corresponds to spectra acquired of InAs and InSb nanowires, respectively, which were normalized to the In $\mathrm{L\alpha_{1/2}}$ emission and subtracted from each other accordingly. (d) Complete spectra as extracted for the edge of the nanowire only at positions above (green - solid), below (blue - solid), at WZ barriers including the QD (red - solid) shown together with reference spectra of InAs (light grey) and InSb nanowires (dark grey) and normalized to the In $\mathrm{L\alpha_{1/2}}$ emission.

c. Type A Overview Conductance plot

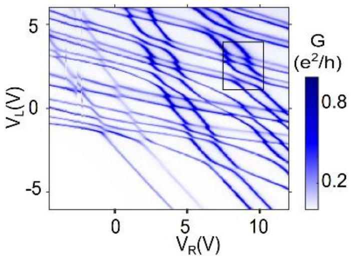

Figure S4: Overview conductance plot as a function of left and right gate voltages for the type-A device shown in figure 2d in the main paper. The rectangular box shows the sharp (6,3)-crossing (top) and the tunnel coupled (5,3)-crossing (bottom).

d. Type B conductance plot and charge stability diagram

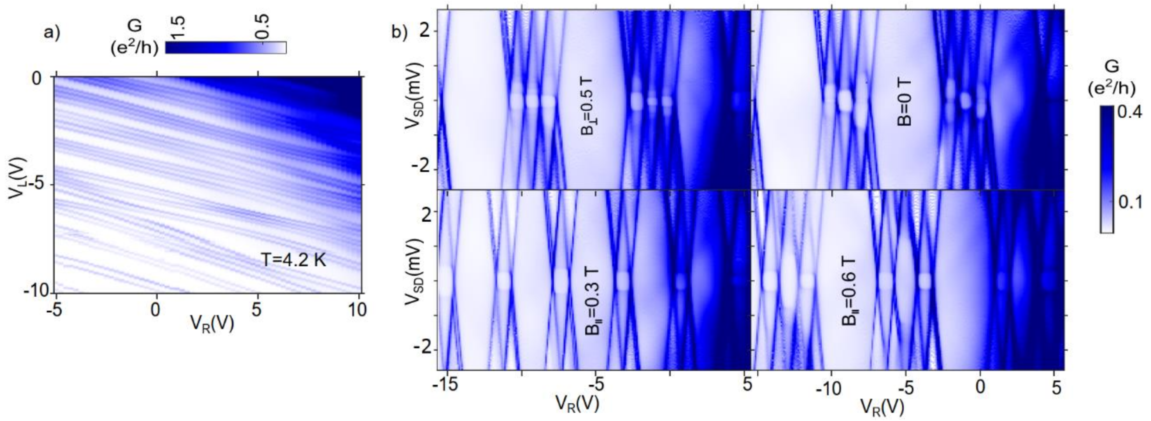

Figure S5: a) Overview conductance plot as a function of left and right gate voltages for a different Type-B sample than in the paper at T = 4.2 K, showing states in groups of four. (Right) Charge stability diagram along the gate vector shown in figure 2a in the main paper at different magnetic fields.

e. Effective chemical potential calculation

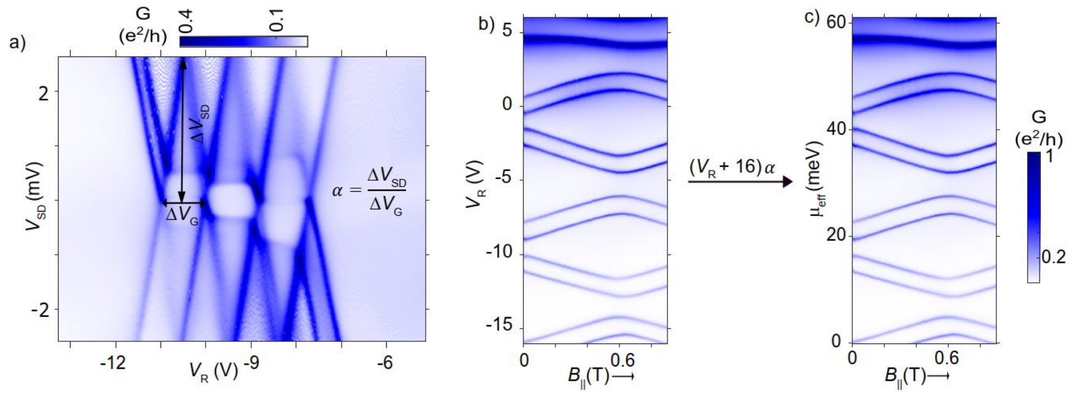

Figure S6: Steps showing the calculation of the effective chemical potential, μ_{eff} a) The gate lever arm (α) is calculated from the charge stability diagram measured along the vector shown in figure 2a in the main paper. The plot of b) right gate, V_R, as a function of $B_{\parallel}$ is converted to c) effective chemical potential by multiplying the y-axis values with α after subtracting the lowest applied V_R (-16 V). A similar method was used for calculating the effective chemical potential for the type-A device.

References

(1) Groth, C. W.; Wimmer, M.; Akhmerov, A. R.; Waintal, X. Kwant: A Software Package for Quantum Transport. *New J. Phys.* **2014**, *16* (6), 063065. https://doi.org/10.1088/1367-2630/16/6/063065.

(2) Potts, H.; Chen, I. –J.; Tsintzis, A.; Nilsson, M.; Lehmann, S.; Dick, K. A.; Leijnse, M.; Thelander, C. Electrical Control of Spins and Giant g -Factors in Ring-like Coupled Quantum Dots. *Nat. Commun. 2019 101* **2019**, *10* (1), 1–7. https://doi.org/10.1038/s41467-019-13583-7.

(3) Dahl, M.; Namazi, L.; Zamani, R. R.; Dick, K. A. Sb Incorporation in Wurtzite and Zinc Blende InAs1−xSbx Branches on InAs Template Nanowires. *Small* **2018**, *14* (11), 1703785. https://doi.org/10.1002/SMLL.201703785.
